# Valorization of Vegetable Processing Residues for Functional Biodegradable Films in Sustainable Food Packaging

**DOI:** 10.3390/foods15152737

**Published:** 2026-08-04

**Authors:** Dewduni Premarathne, Xiaoyang He, Suwimol Chockchaisawasdee, Costas Stathopoulos

**Affiliations:** 1 School of Medical, Molecular and Forensic Sciences, Murdoch University, Murdoch, WA 6150, Australia; dewduni.premarathne@murdoch.edu.au (D.P.); xiaoyang.he@murdoch.edu.au (X.H.); suwimol.chockchaisawasdee@murdoch.edu.au (S.C.); 2 Food Futures Institute, Murdoch University, Nambeelup, WA 6181, Australia

**Keywords:** biopolymers, bioactives, green extractions, film characterization, circular economy

## Abstract

The increasing accumulation of vegetable processing waste presents significant environmental challenges while also offering a sustainable source of structural and functional materials for biodegradable film development. This review summarizes recent progress in the utilization of vegetable by-products, including peels, pomace, stalks, and seeds, as sources of biopolymers and bioactive compounds for biodegradable film production. Extraction techniques, such as ultrasound-assisted, microwave-assisted and enzyme-assisted processes, are discussed to highlight advances in improving the efficiency and sustainability of biopolymer and bioactive recovery. In addition, film development strategies and the effects of incorporating these extracted compounds on mechanical, barrier, antioxidant, and antimicrobial properties of films are critically examined. Overall, this review provides insights into the potential and current limitations of vegetable-waste-derived biodegradable films and outlines future directions to support their development within a circular bioeconomy framework.

## 1. Introduction

Fruits and vegetables are among the most widely consumed commodities [1] due to their rich nutritional value and associated health benefits, such as reduced risk of chronic diseases—including cardiovascular diseases, certain types of cancer, obesity, and type 2 diabetes—and improved digestive health and overall immune function [2,3]. In response to population growth and a global shift toward healthier dietary patterns, the production of fresh produce has increased substantially. In 2023, world fruit and vegetable production reached 2.1 billion tonnes [4]. This expansion has also led to a corresponding rise in processing residues and waste, which has emerged as a significant environmental and economic challenge worldwide [5,6], highlighting the need for sustainable management and valorization strategies [7].

Large quantities of vegetable by-products (VBP), including peels, pomace, seeds, stems, and pulp residues, are discarded despite being rich sources of valuable biopolymers and bioactive compounds [8,9]. Conventional disposal methods, such as landfilling and incineration, not only contribute to environmental pollution but also result in the underutilization of potentially valuable biomass [10]. These disposal methods also give rise to several social concerns. The accumulation of agro-industrial waste can contribute to public health issues through the emission of harmful gases, unpleasant odors, and the proliferation of disease vectors, particularly in communities located near disposal sites [11,12].

Common methods for utilizing fruit and vegetable processing waste typically focus on low-value applications such as animal feed and organic fertilizers, rather than exploiting it to its full potential [13,14]. In recent years, waste valorization has gained significant attention as a sustainable approach to transform agro-industrial residues into higher-value products across multiple sectors, including food, pharmaceutical, cosmetic, energy, and agricultural industries [15,16]. This shift reflects a broader move away from conventional disposal practices towards innovative upcycling strategies that enhance resource efficiency and support the development of a circular bioeconomy [6]. Among emerging valorization strategies, the development of functional biodegradable films from fruits and VBPs has gained attention as a promising and sustainable approach [17,18]. However, compared to fruit-derived residues, VBPs remain relatively underexplored despite their significant potential as sources of functional biopolymers and bioactive compounds. This can be due to the fact that fruit waste (e.g., citrus waste, apple pomace, and grape pomace) is highly centralized due to the massive global juice and wine industries. This centralized stream has naturally drawn the bulk of academic and industrial valorization research.

Vegetable processing residues contain abundant polysaccharides such as pectin, cellulose, hemicellulose, and starch, which can serve as renewable film-forming materials [19,20]. In addition, these by-products are rich in natural antioxidants, antimicrobial compounds, pigments, vitamins, and phenolic substances that can impart functional properties to biodegradable films, enabling the development of active packaging materials capable of extending shelf life, enhancing food quality, and reducing microbial spoilage [21,22].

In addition to the benefits of minimizing agro-industrial waste accumulation, the potential of VBPs for biodegradable film production also lies in their ability to offer multiple environmental and economic advantages, including reducing dependence on petroleum-based plastics and promoting the sustainable use of renewable resources in alignment with global sustainability goals [23]. This approach is closely aligned with the United Nations Sustainable Development Goals (SDGs), particularly SDG 2 (Zero Hunger), SDG 12 (Responsible Consumption and Production), and SDG 13 (Climate Action), as emphasized by the Food and Agriculture Organization (FAO), which advocates for the reduction in food loss and waste and the efficient utilization of agricultural resources [24].

As previously highlighted, research on biodegradable films derived specifically from VBPs remains relatively limited compared to studies focusing on fruit residues. To the best of our current knowledge, a dedicated review that addresses biodegradable film development exclusively from VBPs remains a distinct gap in the literature. In addition, the development of functional biodegradable films that incorporate bioactive compounds to provide antioxidant, antimicrobial, or intelligent packaging properties from vegetable-derived residues is still in its early stages. These gaps highlight the need for more focused research to fully exploit the potential of VBPs as sustainable sources of both structural and functional materials for biodegradable films.

Therefore, this review focuses on the potential of VBPs as sources for the development of functional biodegradable films. The review discusses the major types of vegetable processing residues suitable as raw materials, the extraction and recovery of biopolymers and bioactive compounds, film-processing techniques, and the functional properties of the developed films. Unlike conventional reviews that primarily focus on purified biopolymers or generalized biomass sources, this work specifically emphasizes vegetable processing residues as integrated film-forming systems, considering both direct utilization of whole biomass and extracted components. In addition, the characterization of these films, current challenges, and future perspectives associated with the industrial application of vegetable by-product-derived biodegradable films are highlighted in the context of sustainable food packaging and circular bioeconomy development.

## 2. Potential of Vegetable By-Products as Sustainable Resources for Biodegradable Films

The valorization of VBPs into biodegradable films is primarily based on the recovery of naturally occurring biopolymers, including polysaccharides and minor protein fractions present in peels, pomace, pulp residues, stems, and seeds. These components serve as fundamental building blocks for the development of sustainable film-forming materials [19,25]. Polysaccharides, including cellulose, residual starch, and pectin, represent the dominant fraction in most VBPs, such as potato peels, carrot pomace, and tomato pomace and are the primary contributors to film formation [26], since these polysaccharides are capable of forming cohesive and continuous matrices through intermolecular interactions such as hydrogen bonding, which enables the development of stable film networks [27]. Within this structural framework, different polysaccharides contribute distinct functional roles. Pectin contributes excellent gelling and film-forming properties [28], while cellulose fibers enhance tensile strength and structural stability of the films [29]. Starch, owing to its abundance and ability to gelatinize, is widely used as a low-cost, biodegradable film-forming polymer in vegetable waste-based systems [30,31,32]. The hydroxyl-rich structure of these polysaccharides facilitates plasticization using compounds such as glycerol and sorbitol, which reduce intermolecular forces and improve flexibility, thereby enabling the formation of uniform and processable film matrices [33,34]. Certain vegetable wastes also contain small amounts of lipids. For example, tomato seeds and pumpkin seeds are, primarily, composed of triacylglycerols rich in unsaturated fatty acids such as linoleic and oleic acids. These lipid components include minor bioactive compounds such as tocopherols and phytosterols, contributing to their functional and hydrophobic properties [35].

Biodegradable films derived from VBPs can be produced using different film-forming techniques. Solvent casting is the most widely used method at the laboratory scale, where film-forming solutions containing extracted biopolymers and plasticizers are cast and dried at low temperature to form uniform films [36,37]. In contrast, the extrusion method involves the application of heat and pressure to form films from solid or semi-solid matrices, offering advantages for scalability and industrial processing. The choice of method significantly influences the structural, mechanical, and barrier properties of the resulting films, as processing conditions can affect polymer interactions, film homogeneity, and overall performance [37]. On an industrial manufacturing scale, the technology closest to commercialization in biodegradable filmmaking is melt-processing extrusion modes. Unlike the slow solvent casting methods commonly used at the research scale, these dry-formation processing modes integrate seamlessly into the high-throughput machinery already standard across the global plastics packaging industry [38,39]. Film-forming solutions can also be applied directly on food matrices as edible coatings, which are thin, consumable layers formed on food surfaces to preserve quality and extend shelf life [40].

Overall, VBPs represent a valuable and underutilized biomass resource, particularly for biodegradable film production. Their intrinsic composition enables the development of packaging materials that combine structural performance and enhanced functionality with environmental sustainability.

As shown in Table 1, most developed films have been evaluated for mechanical and barrier properties, while their application in real food systems remains limited, highlighting a gap between laboratory characterization and practical use. One challenge for the real-world application of plant extracts is that they can interfere with water resistance because they often contain hydrophilic compounds such as sugars, organic acids, and phenolics that increase water absorption and disrupt the structural integrity of the polymer matrix. This may explain the hydrophilic behavior observed in the film developed by Choque-Quispe et al. [41], who incorporated carrot extract into a potato starch matrix. Similar results were reported by Cvetković et al. [42], where the incorporation of wild blackberry extract into starch-based films significantly increased water content, swelling, and water-vapor permeability due to the hydrophilic nature of the extract compounds. Perotto et al. [43] researched how water affects vegetable-based bioplastic films. They explained that water absorption can act as a plasticizing agent in vegetable-based bioplastic films by reducing intermolecular interactions between polymer chains and increasing chain mobility, resulting in decreased stiffness and increased flexibility. This further explains the hydrophilic behavior of vegetable-based films.

**Table 1:** Comparison of biodegradable films incorporating vegetable by-products.

Vegetable By-Products	Processing Method	Film Composition	Key Properties	Reference
Beetroot peel	Solvent casting	Chitosan, glycerol, PVA, beetroot peel extract	Beetroot peel extract functions as a pH indicator and antimicrobial agent. TS—3.70 ± 0.06 MPa, EB —96.08 ± 0.31%, YM—3.80 ± 0.01 MPa.	[44]
Potato peel	Solvent casting	PPS, glycerol	PPS increments decrease water solubility, swelling power, water permeability, and display good mechanical properties. TS—9.23–13.95 MPa, EB—15.04–22.65%.	[31]
Potato peel	Acid hydrolysis, solvent casting	PPS, glycerol	The combination of mild acid hydrolysis and plasticization allows the development of homogeneous, transparent, and flexible films. TS—12.7 MPa, EB—18.4%.	[45]
Potato discards	Solvent casting	PS, glycerol, ethanolic propolis extract	The films inhibit the growth of *Staphylococcus aureus.* Propolis improves moisture barrier performance. Films fully degrade in 30 days. TS—2.15–5.15 MPa, WVP—0.44 × 10^−10^ to 8.55 × 10^−10^ g/(m·s·Pa).	[32]
Potato and carrot discards	Solvent casting	PS, cactus mucilage, carrot extract, citrus pectin, glycerin	Carrot extract improves the functional properties of the films. Films have moderate strength and high flexibility. Strongly hydrophilic properties. TS—4.5–5.7 MPa, EB—87–93%, WVP—0.9 × 10^−2^ to 1.6 × 10^−2^ g mm/h m^2^ Pa.	[41]
Pumpkin seeds and peels	Ultrasound treatment, solvent casting	DPS, PP, soy lecithin, glycerol, CaCl_2_	DPS:PP ratio of 1:1 offers a balance of strength, flexibility, good tensile strength, elongation, and barrier performance. TS—1401 ± 5.4 kPa, EB—9.74 ± 0.46%, WVP—8.79 ± 0.12 × 10^−6^ g/Pa m h. Ultrasound treatment enhances film casting and properties.	[46]

PVA—polyvinyl alcohol; PPS—potato peel starch; PS—potato starch; DPS—defatted pumpkin seed; PP—pumpkin peel powder; TS—tensile strength; EB—elongation at break; YM—Young’s modulus; WVP—water vapour permeability.

The influence of plant-derived additives on moisture resistance is highly system-dependent and often contradictory, reflecting differences in extract composition, matrix compatibility, and structural organization. For example, the addition of *Moringa stenopetala* leaf extract reduced moisture resistance, as evidenced by an increase in the WVP of sweet potato starch-based films [47]. Similarly, the incorporation of beetroot waste extract into edible films developed with *Opuntia ficus-indica* mucilage was reported to increase the WVP, which was attributed to the hydrophilic characteristics of betalains [48]. In contrast, the addition of ethanolic propolis extract has been reported to improve the moisture barrier properties [49,50]. This high-water resistance is due to its composition, which is rich in hydrophobic resins, waxes, and lipophilic compounds, and it is identified as a good addition to biodegradable films [51].

Surface coating of plant-based films is another way to improve their water resistance. Surface modification by coating films with ZnO nanoparticles to produce superhydrophobic surfaces and increase the water contact angle has been proposed as a novel approach to further enhance moisture barrier properties in biodegradable films [52]. This approach could also be applied to vegetable waste-based films because ZnO is currently listed as a generally recognized as safe (GRAS) material by the Food and Drug Administration [53]. Apart from providing hydrophobicity, ZnO can act as an antimicrobial agent due to its activity against a wide range of microorganisms when incorporated into polymer matrices and has high resistance to processing conditions [54].

Beyond their role as film base materials, the incorporation of vegetable by-product-derived materials into film formulations has also been shown to enhance functional properties. Natural compounds such as polyphenols and flavonoids present in plant residues can act as antioxidants and antimicrobial compounds [55,56]. For example, beetroot peel is a rich source of betalains and polyphenols. Betalains change color based on acidity, where they remain red/pink in acidic conditions (pH 3–4), turn brown as they become neutral, and shift to yellow-green in alkaline conditions [44]. Betalains are unstable in the presence of light and oxygen and are degraded when subjected to high temperatures [57]. This should be carefully considered during smart film preparation to preserve the functional properties of the pigments, because during film casting and drying, elevated temperatures and prolonged exposure to air can promote oxidative degradation and structural breakdown of the pigment molecules [58]. High drying temperatures and prolonged drying times can accelerate pigment degradation through oxidation and structural decomposition, thereby reducing colour intensity and antioxidant activity. Consequently, mild drying techniques, such as low-temperature air drying, vacuum drying, or freeze-drying, are often preferred to improve betalain retention [59]. Storage conditions can significantly influence pigment stability. Exposure to light, oxygen, elevated temperatures, and high relative humidity during storage can further decrease betalain content, leading to colour fading and reduced functionality of smart packaging films. Therefore, appropriate drying parameters and protective storage conditions are essential for maintaining the stability and performance of betalain-containing biodegradable films throughout their shelf life [60,61].

Therefore, many VBPs present significant potential for further research in eco-friendly packaging solutions, as they are low-cost, widely available, and rich in biopolymers and bioactive compounds, enabling the development of functional films [62,63]. Their utilization also supports zero-waste and circular economy approaches by converting agro-industrial residues into value-added materials and minimizing overall waste generation [5,60]. To effectively utilize these underexploited resources in biodegradable packaging applications, it is essential to isolate and recover their key functional components.

## 3. Recovery of Functional Components from Vegetable By-Products

### 3.1. Extraction of Biopolymers from Vegetable By-Products

Biopolymers form the basic structure of biodegradable films, since they form the matrix or continuous structure [61]. VBPs represent an abundant and sustainable source of biopolymers, including polysaccharides (cellulose, hemicellulose, and pectin), proteins, and minor lipid fractions, which can be effectively recovered for use in biodegradable and functional materials [64] with affordability, which may address the high-cost concern about the practical application of biopolymers [65].

Natural polymers offer significant environmental advantages, particularly due to their inherent biodegradability, which makes them an eco-friendly choice for packaging applications [66]. The extraction of these biopolymers typically involves a combination of mechanical, chemical, and emerging green techniques designed to disrupt complex plant cell wall structures and release target macromolecules [67,68]. Conventional extraction techniques, such as acid and alkaline treatments, are widely applied for isolating biopolymers such as cellulose and pectin [69,70]. Acid extraction, typically using diluted mineral acids (e.g., hydrochloric or sulfuric acid), is widely used for pectin recovery, where acidic conditions promote the solubilization of pectic substances from the plant cell wall. The industrial acid extraction process involves a relatively long process (up to several hours) performed at low pH (1–3) and increased temperatures (50–100 °C) [71]. Therefore, these approaches are often associated with high energy and time requirements, and they may pose environmental challenges due to the generation of acidic waste streams and the corrosion of processing equipment [72]. Alkaline extraction, using solutions such as sodium hydroxide, is primarily applied for cellulose and hemicellulose isolation by removing lignin, proteins, and other non-cellulosic components, thereby facilitating the release of structural polysaccharides [26,73]. Alkali-based methods have also been applied for pectin extraction, but these processing conditions lead to partial degradation of pectin structure [74]. To address these limitations, greener extraction strategies—including enzyme-assisted extraction (EAE), ultrasound-assisted extraction (UAE), deep eutectic solvents (DES), ionic liquids (ILs), subcritical and supercritical extraction, and microwave-assisted extraction (MAE)—have gained increasing attention [68,75]. Enzymes have the capacity to perform highly selective extraction or modification of biopolymers under mild conditions. They selectively hydrolyze structural components such as starch (by means of amylases) and pectin (by means of pectinase), improving the yield and purity of extracted biopolymers while preserving their functional properties [68]. UAE and MAE enhance mass transfer, reduce extraction time, and lower solvent consumption, making them more energy-efficient alternatives [67]. The efficiency of biopolymer extraction is strongly influenced by process parameters such as pH, temperature, extraction time, and solvent composition, which must be carefully optimized to balance yield, structural integrity, and functionality [68].

Despite these advantages, challenges related to variability in raw material composition, scalability of green extraction methods, and consistency of quality of extracted biopolymers remain key areas requiring further research [75]. A fundamental trade-off exists between maximizing extraction yield and preserving polymer functionality. Maximizing yield typically requires harsh conditions that degrade the polymer structure, while preserving functionality requires mild methods that limit recovery. High temperatures, extreme pH levels, and prolonged extraction times maximize the breakdown of cellular matrices, leading to higher yields [76,77]. However, these factors cause polymer chain scission, structural degradation, and the loss of thermo-labile functional groups. For example, Lasunon and Sengkhamparn [78] reported that harsh conditions, such as high acidity and the frequency of sound waves, caused the degradation of pectin structure during the extraction of pectin from industrial tomato waste. Similar results were observed by Van Audenhove et al. [79], where acid extraction under harsh conditions degraded pectin structure while increasing extraction efficiency. Sensitive biological properties, such as antimicrobial, antioxidant, or enzyme-inhibitory activities, rely on intact chemical structures. Harsh extraction techniques often inactivate or alter these reactive sites [80].

Studies summarized in Table 2 demonstrate that VBPs are promising sources of valuable biopolymers, particularly pectin and cellulose-based materials. A notable trend across the studies is the increasing reliance on integrated and emerging extraction techniques, including enzymatic treatment combined with ultrasonic pretreatment, microwave-assisted extraction, and high-pressure homogenization, which consistently outperform conventional methods in terms of yield and efficiency. Enzyme mixtures were found to be more effective in obtaining higher yields than single enzymes in pectin isolation from beet pulp, highlighting the importance of synergistic mechanisms in degrading complex plant cell wall matrices [81]. Enzyme extraction prevents the neutralization and purification of pectin that is necessary after conventional acid extraction methods. The combination of ultrasonication and high-pressure homogenization enabled the successful production of cellulose nanofibers from potato residues, indicating the potential of mechanical-assisted processes for generating high-value nanostructured biopolymers [82]. Optimization of process parameters using Response Surface Methodology (RSM) was found to increase the extraction yield of MCC from carrot pomace [83]. Similarly, RSM was applied to optimize extraction conditions during pectin extraction from pumpkin peels [84]. These findings highlight the effectiveness of RSM in maximizing the extraction efficiency of biopolymers from vegetable residues with respect to the extraction yield. However, most studies prioritize extraction yield and physicochemical characteristics, while overlooking how structural modifications induced by processes such as ultrasonication or microwave heating influence film-forming ability, barrier properties, and mechanical strength. Cui and Zhu [85] reported that the structure of polysaccharides was changed after ultrasound processing, leading to modifications of their physicochemical and biological properties. Similarly, Zhu et al. [86] reported on ultrasound-induced changes in the structure and functionality of starch and protein, highlighting the need to optimize ultrasound parameters in future research studies. While VBPs represent a highly promising and sustainable source of biopolymers, future research should prioritize the development of scalable, environmentally friendly extraction processes that balance high recovery efficiency with the preservation of functional properties.

**Table 2:** Biopolymers Derived from Vegetable By-products: Comparison of Extraction Methods and Key Findings.

Vegetable By-Products	Extracted Compounds	Extraction Method	Key Findings	Reference
Beet pulp	Pectin	Ultrasonic-assisted enzymatic extraction	The use of an enzyme mixture was more effective in pectin isolation than single enzymes. Use of ultrasonic pretreatment before the enzymatic isolation of pectin was effective in decreasing the time for obtaining the highest possible yield under the conditions used.	[81]
Potato residues after starch processing	Cellulose nanofibers (CNF)	Ultrasonication combined with high-pressure homogenization	The mechanical force by ultrasonication combined with high-pressure homogenization caused the cellulose in the potato residues to be broken into CNFs without affecting the chemical structure of cellulose.	[82]
Tomato pomace	Pectin	Acid hydrolysis, aqueous extraction	The yield and the purity of the extract obtained by the acid method were significantly higher than those of the sample obtained by the aqueous one.	[87]
Carrot pomace	Microcrystalline cellulose (MCC)	Autoclave and ultrasound-assisted extraction	Higher yields represented a more environmentally sustainable approach compared to the conventional acid hydrolysis method.	[83]
Black carrot pomace	Pectin	Microwave, ultrasound, and conventional heating	The yield was found to be directly correlated with the degree of esterification, galacturonic acid content, particle size, and water holding capacity. Microwave extraction was observed to exhibit the highest rate of extraction due to its instantaneous volumetric heating ability. Ultrasound extraction resulted in polymer breakdown, and therefore a smaller particle size and lesser gelling capabilities were evident.	[88]
Pumpkin peel	Pectin	Microwave-assisted extraction	The pH was a crucial parameter affecting pectin extraction yield, with lower pH values significantly enhancing the extraction yield due to the hydrolysis of polysaccharides under strongly acidic conditions.	[84]
Eggplant peel	Pectin	Ultrasonic extraction	Extracted pectin showed good functional features such as water holding capacity and oil holding capacity, emulsifying and foaming properties, and antioxidant activity.	[89]

Despite their environmental advantages, green extraction technologies are often constrained by significant economic limitations that hinder their large-scale industrial adoption [90]. High initial capital investment remains a major barrier, particularly for advanced systems such as ultrasound, microwave, and high-pressure-assisted extraction [91]. In addition to capital costs, operational expenditures can be substantial due to energy requirements, maintenance, and the need for skilled labor [92,93]. Among green techniques, EAE presents a distinct economic challenge due to the high cost of enzymes, which can represent a dominant fraction of total operating expenses [94]. Supercritical fluid extraction (SFE) is associated with high capital investment costs, which represent a major barrier to its industrial implementation. The requirement for specialized high-pressure equipment, including extraction vessels, compressors, and pressure-resistant infrastructure, significantly increases the initial investment [95].

### 3.2. Extraction of Bioactive Compounds from Vegetable By-Products

Bioactive compounds derived from plant sources have attracted considerable attention due to their diverse health-promoting properties and wide range of industrial applications, including pharmaceuticals, cosmetics, and food and beverages [96,97]. VBPs, generated in large quantities during processing, represent inexpensive and abundant raw materials that are rich in valuable bioactive constituents, such as phenolics, carotenoids, and dietary fibers [60,98]. The incorporation of these compounds into biodegradable film matrices offers a promising strategy to enhance the functional performance of packaging materials. Such films can exhibit improved antioxidant and antimicrobial properties, thereby extending shelf life, maintaining food quality, and contributing to the development of sustainable and active packaging systems [99,100]. The inclusion of bioactive compounds, such as natural pigments, contributes to the development of smart films capable of indicating changes in food freshness or environmental conditions [101].

Conventional extraction of bioactive compounds includes maceration, cold pressing, steam distillation, and Soxhlet’s extraction [102]. Conventional extraction methods often exhibit relatively low extraction efficiencies, requiring multiple extraction cycles to achieve satisfactory yields. The low extraction efficiency results in increased processing time, higher energy consumption, and solvent usage, ultimately reducing the overall sustainability of the process [96]. Modern green extraction technologies such as MAE, UAE, and EAE offer significant advantages in reducing processing times, minimizing hazardous solvent waste, and preserving heat-sensitive molecules [96,103].

The recovery of high-value bioactive compounds from VBPs requires selecting an extraction method that balances high yield with environmental and economic considerations. Effective extraction techniques ensure optimal resource utilization and reduce secondary waste generation by maximizing yield. The following section evaluates the performance and operational parameters of both conventional and emerging extraction methods to identify the most viable pathways for transforming VBPs into functional industrial ingredients.

The data summarized in Table 3 demonstrate that the type of extracted bioactives varies significantly depending on the raw material, with carotenoid-rich matrices predominantly observed in carrot, tomato, and pumpkin by-products, while phenolic compounds are more abundant in beetroot, cauliflower, and cabbage residues. The aforementioned studies have used different extraction techniques to extract bioactive compounds from VBP. Major bioactive compounds extracted are carotenoids (β-carotene, α-carotene, lycopene, and lutein) and phenolics [98]. In many studies, UAE and MAE have been effectively applied to VBPs to optimize carotenoid recovery, as shown in Table 3. These novel methods have improved the extraction yield compared to conventional solvent-based methods, while also reducing extraction time and improving efficiency. In UAE, acoustic cavitation generates microbubbles that collapse violently, producing localized high pressure and temperature, which rupture cell walls and increase solvent penetration into the plant matrix. This facilitates the rapid release of intracellular bioactive compounds into the solvent [104,105]. Similarly, MAE uses microwave energy to heat polar molecules within the sample and solvent, causing rapid internal heating and pressure build-up inside plant cells. This leads to cell wall disruption and improved diffusion of target compounds. Both techniques reduce extraction time, lower solvent consumption, and improve yield compared to conventional methods, making them highly efficient for recovering bioactive compounds from plant-based materials, such as VBPs [106,107]. Supercritical fluid extraction (SFE) is an advanced separation technique that utilizes a fluid above its critical temperature and pressure to selectively extract target compounds from solid or liquid matrices. In the context of VBP, supercritical carbon dioxide (CO_2_) is most commonly employed due to its non-toxicity, low critical temperature, and ease of removal after extraction. This method is particularly effective for recovering non-polar and moderately polar bioactive compounds, such as carotenoids (e.g., lycopene and β-carotene) from tomato waste, carrot pomace, and pumpkin residues [108,109].

**Table 3:** Comparison of bioactive compound extraction using different extraction methods.

Vegetable By-Products	Extracted Compounds	Extraction Method	Extraction Solvent/ Medium	Key Findings	Reference
Carrot pomace	Carotenoids	UAE, high shear dispersion technique	Flaxseed oil	Solvent-free extraction using flaxseed oil and ultrasonication.	[110]
Carrot pomace	Carotenoids, phenolics	Ultrasound pretreated extraction, enzymatic pretreated extraction, conventional extraction	Ethanol, hexane, ethanol–hexane mixture	Enzymatic pretreatment with pectinase is the most effective compared to conventional solvent extraction.	[111]
Carrot pomace	Phenolics	UAE	Ethanol	Optimum conditions for UAE are 10 min and 250 W of ultrasonic power in 70% ethanol.	[112]
Carrot pomace, including rejected carrots	Carotenoids, phenolics	MAE, UAE, CSE	Hexane and ethanol	MAE recovers the maximum yield of bioactive components (78% dry basis) compared to UAE and CSE.	[113]
Beetroot pomace	Phenolics	PLE.	Ethanol, ethanol–distilled water mixture	PLE enables the recovery of high amounts of phenolic compounds from beetroot residues.	[114]
Tomato skin	Lycopene	Supercritical fluid extraction	Supercritical carbon dioxide fluid	The solubility values of lycopene increase with both pressure and temperature.	[108]
Tomato processing waste (skin and seeds)	Lycopene, β-carotene, lutein	Solvent extraction	Hexane, acetone, ethanol, ethyl acetate, and ethyl lactate	Ethyl lactate (a green solvent) is highly effective for extracting carotenoids from tomato waste, significantly outperforming other tested organic solvents.	[115]
Eggplant peel	Nasunin	Acid extraction	HCl	Antioxidant solutions were made with semi-purified extracts depleted of chlorogenic acid or with purified anthocyanin crystals. The semi-purified nasunin extract was higher in scavenging capacity.	[116]
Cauliflower and cabbage waste	Phenolics, flavonoids	Solvent extraction	Ethanol	Cauliflower waste yielded higher Total Phenolic Content and Total Flavonoid Content compared to cabbage.	[117]
Pumpkin by-products	Carotenoids	Supercritical CO_2_ (SCCO_2_), Soxhlet extraction	SCCO_2_, n-hexane, ethanol, vegetable oil (rapeseed, coconut, grapeseed, and olive oil)	SCCO_2_ extraction recovered the highest volume of total carotenoids (TCC) from pumpkin seed extracts. Among the edible vegetable oils evaluated, olive oil contained the highest initial total carotenoids.	[109]

Ultrasound-assisted extraction—UAE; microwave-assisted extraction—MAE; conventional solvent extraction—CSE; pressurized liquid extraction—PLE.

The effectiveness of the extraction process is strongly influenced by parameters such as extraction time, solid-to-solvent ratio, temperature, pH, and particle size of the raw material. These factors collectively determine not only the extraction yield, but also the chemical profile and antioxidant potential of the obtained extracts [96].

Studies indicate that increasing extraction time and temperature can enhance solute solubility and accelerate mass transfer, thereby improving extraction performance. Nonetheless, excessive conditions may lead to the degradation of heat-sensitive compounds, particularly phenolic constituents [118]. Longer extraction periods can promote the degradation of polyphenols through increased exposure to oxygen and light [119]. Lower solid-to-solvent ratios are typically associated with enhanced extraction yields due to improved mass transfer. However, excessively low ratios can lead to increased solvent consumption and longer downstream concentration steps [96].

In addition to extraction techniques, solvent selection is a critical factor influencing both extraction performance and environmental sustainability. In particular, the solvent’s polarity plays a key role in determining the selective recovery of different classes of bioactive compounds [120]. While conventional organic solvents such as hexane and acetone remain effective, there is a clear shift toward greener alternatives. Ethanol is widely used due to its food-grade status and low toxicity, whereas ethyl lactate has emerged as a particularly effective green solvent, significantly outperforming other solvents in the extraction of carotenoids from tomato waste [115]. Due to concerns associated with conventional solvent extraction, such as the use of flammable chemicals and the potential toxicity of residual by-products, there is growing interest in using edible oils as an alternative in extraction methods, particularly those that are more environmentally sustainable [121]. Flaxseed oil has been shown to be an effective solvent for carotenoid extraction from carrot pomace [110]. It has also been reported that the refining process of vegetable oils can influence and potentially reduce the extraction yield. Therefore, the impact of oil refining should be carefully considered in future studies when evaluating vegetable oils as extraction media for carotenoids [109].

Despite these advances, the comparative environmental impacts of these solvents are rarely quantified using life-cycle assessment approaches, limiting the ability to make fully informed decisions regarding sustainable solvent selection [122], thereby hindering industrial adoption. In addition, solvent residues and their potential effects on downstream applications, particularly in food-contact materials, remain insufficiently investigated and are rarely validated in real food systems [123]. Pre-treatment methods further contribute to improved extraction performance. Techniques such as enzymatic, high-pressure processing, and ultrasound pre-treatments enhance the disruption of plant cell matrices, facilitating the release of bound bioactive compounds [124]. A key benefit of enzymatic hydrolysis is its ability to lower the activation energy required for chemical reactions, allowing the process to occur under milder and more controlled conditions [110]. However, the cost and stability of enzymes, along with potential variability in enzyme activity depending on substrate composition, remain challenges that need to be addressed for large-scale implementation [125]. High-pressure processing is recognized as an effective green technology for disrupting plant and microbial cell structures prior to the extraction of bioactive compounds, and it enhances mass transfer and improves extraction efficiency while avoiding the use of high temperatures and harmful solvents [126].

The recovery of these bioactive compounds from VBPs has significant implications for the development of functional biodegradable materials [25]. These bioactive compounds can impart antioxidant properties to films, thereby enhancing their functional performance, improving oxidative stability, and potentially extending the shelf life of packaged food products [127]. However, the stability of these bioactives during film processing (e.g., drying, heat exposure, and storage) is often insufficiently investigated, which may limit their practical effectiveness in real food systems [128]. Additionally, the compatibility of extracted compounds with different film-forming matrices and their influence on mechanical and barrier properties require more systematic evaluation. Green extraction technologies such as UAE and MAE still face several limitations, including high solvent viscosity, mass transfer resistance, limited selectivity toward certain bioactive compounds, potential degradation of thermolabile constituents, high equipment and operational costs, and challenges associated with process optimization and industrial scalability [129,130].

Future research should prioritize process scalability, economic feasibility, and environmental impact assessment, while also addressing the stability and functionality of the recovered bioactives in end-use applications. Such efforts are essential to bridge the gap between laboratory-scale findings and industrial implementation, thereby enabling the development of truly sustainable and functional materials within a circular economy framework.

## 4. Development of Functional Biodegradable Products Derived from Vegetable By-Products

Since VBPs are rich sources of biopolymers and bioactive compounds, they provide two main approaches for biodegradable film development: extraction of biopolymers for use as the film-forming matrix and direct utilization of whole pomace for film production. The former has been extensively studied, whereas the latter has received comparatively less attention. Utilizing the whole raw material offers a practical strategy to minimize incompatibility issues among different biopolymers, as it avoids the need for separate extraction and recombination processes [131]. Certain factors such as high instability and moisture content, followed by high dehydration and processing costs, limit the use of whole pomace [132]. Pakulska et al. [133] mentioned that the addition of fruit pomace powder did not form a continuous film structure, highlighting a key limitation of directly using whole pomace in film formation. This observation may indicate a broader limitation of directly using whole pomace, which could also be relevant to vegetable pomace-based film systems. Both approaches provide opportunities for sustainably valorizing food processing waste and developing environmentally friendly packaging material.

The studies summarized in Table 4 illustrate the functional biodegradable films derived from VBP. Most of the developed films rely on the incorporation of externally added bioactive agents, particularly essential oils and plant-derived extracts, to impart functional properties such as antioxidant and antimicrobial activity. A significant research gap remains in the limited utilization of the bioactive compounds naturally present in VBP. The table also highlights the frequent use of additional synthetic polymers, plasticizers, and stabilizers, including polyvinyl alcohol, chitosan, glycerol, sorbitol, and surfactants, indicating that fully vegetable by-product-derived film systems are still scarce. The compatibility of the added ingredients with the film matrix plays an important role in the final film properties. Xie et al. [131] reported that bacterial cellulose had a promising compatibility with the potato peel matrix compared to plant cellulose. They reported that the interactions between bacterial cellulose and the potato peel matrix via hydrogen bonding led to a decrease in the number of hydrophilic free hydroxyl groups, and thus a decrease in Water Vapor Permeability (WVP) of the potato peel films. Kang and Min [134] reported that films derived from potato peel displayed high hydrophilicity. To address this issue, Borah et al. [65] incorporated sweet lime pomace into the film matrix, which effectively reduced hydrophilicity and improved the overall film properties. Addition of vegetable proteins was also found to contribute to a reduction in water solubility, enhancing the water resistance of the bio-composite films [135].

**Table 4:** Functional biodegradable films derived from vegetable by-products.

Vegetable Waste Sources	Film Composition	Bioactive Agents	Functional Properties	Reference
Potato peel	Potato peel powder, bacterial cellulose, glycerol	Curcumin	Antioxidant capacity, prevention of lipid oxidation	[131]
Potato peel	Potato peel powder, sweet lime pomace, glycerol, egg yolk, clove essential oil	Clove essential oil	Antimicrobial activity against *Escherichia coli*	[65]
Vegetable pomace (carrot and beetroot juice extract)	Cellulose nanofibres from vegetable pomace, chitosan, polyvinyl alcohol, and copper oxide nanoparticles from onion peel extract	Copper oxide nanoparticles from onion peel extract	Antimicrobial activity against *Streptococcus, Pseudomonas, Escherichia coli*, and *Klebsiella*	[136]
Cassava bagasse	Cassava bagasse powder, glycerol, sorbitol, coriander essential oil, Tween 20	Coriander essential oil	Antimicrobial activity against *Escherichia coli* and *Streptococcus aureus* (tested on fresh grapes)	[137]
Cassava peel starch	Cassava peel starch, gelatin, amla pomace extract, glycerol	Amla pomace extract	Antimicrobial activity (tested on palmyrah fruit leather)	[138]
Carrot pomace	Carrot pomace, wheat gluten, zein, ethanol, NH_3_ solution	Polyphenols	Antioxidant activity	[139]

The method of incorporating bioactive compounds plays a significant role in enhancing final film properties, particularly in determining their distribution, stability, and overall functional performance [128]. For example, Sothilingam et al. [138] used spray-dried microcapsules of amla pomace extract and reported enhanced antimicrobial activity, which effectively reduced microbial growth on palmyrah fruit leather during 50 days of storage. A direct blending approach was used by Chhoden et al. [139] to incorporate black carrot pomace rich in anthocyanins and polyphenols into a film-forming solution. A similar approach was followed by Singh et al. [136] and Xie et al. [131]. This method is among the most widely used techniques for incorporating bioactive compounds into film-forming systems due to its simplicity and ease of application. Direct blending offers simplicity, low cost, and ease of application, but suffers from poor stability of bioactive compounds, uneven distribution, and rapid release, limiting its effectiveness for long-term functionality [128]. In addition to these methods, nanoemulsion-based encapsulation, coating, and layer-by-layer casting can be applied to incorporate bioactive compounds into films successfully [140,141]. Encapsulation techniques, such as nanoemulsions and microcapsules, provide enhanced protection of sensitive compounds, improved dispersion, and controlled release, although they involve higher processing complexity and cost [142,143]. Layer-by-layer approaches allow precise localization of bioactives and improved functional performance but may face challenges related to adhesion and scalability [144]. Considering all the factors, encapsulation-based methods are the most suitable for incorporating bioactive compounds into vegetable by-product-based films, as they offer the best balance between stability, functionality, and controlled delivery, which are critical for developing high-performance biodegradable packaging systems. The characteristics of the bioactive components, the properties of the matrix, and the conditions of the surrounding release media [145]. Storage conditions such as exposure to oxygen, light, moisture, and temperature can lead to degradation and reduced functional activity of bioactive compounds [146]. Therefore, optimizing bioactive–polymer interactions is essential to maintain antioxidant and antimicrobial effectiveness throughout the packaging application.

The use of indigenous bioactive materials has the potential to limit the need to incorporate externally sourced or isolated bioactive compounds during film preparation, thereby reducing production costs [128]. Given that VBPs are naturally rich in bioactive constituents, their utilization can provide a cost-effective approach for the development of functional biodegradable films. Recent advancements in emerging fabrication strategies—including Pickering’s emulsion-based stabilization, 3D printing, and artificial intelligence (AI)-assisted material design—offer promising opportunities to overcome the limitations of biodegradable films [147,148,149]. Essential oils, which possess strong antioxidant and antibacterial activities, are widely used in Pickering’s emulsion systems to improve their compatibility and controlled release within polymer matrices [150]. AI-driven approaches facilitate more intelligent formulation, performance prediction, design optimization, and sustainability analysis of biodegradable materials [151]. Accordingly, 3D printing enables the development of customized packaging structures with improved functionality [148]. Although applications of these advanced technologies in vegetable by-product-derived biodegradable films remain largely unexplored, their integration may provide new pathways to enhance mechanical performance, stability, and functional properties of such sustainable packaging materials.

Inadequate thermal stability poses a formidable challenge when scaling laboratory-developed biodegradable films to industrial-scale production [152]. While laboratory-scale synthesis relies on slow solvent casting under low-temperature drying profiles, industrial processing requires high-shear melt extrusion. Consequently, lab-optimized formulations often suffer from thermal degradation, burning, or a complete loss of structural integrity when subjected to the high temperatures and intense shear fields of factory equipment. This processing vulnerability is aligned with findings by Goetjes et al. [153], who observed that intense industrial shear fields and high temperatures cause thermal and hydrolytic degradation in PLA/potato starch matrices, systematically undermining the physical parameters of the composite films. Industrial processing equipment requires highly standardized raw materials to maintain uniform film thickness and structural density. However, raw VBPs exhibit continuous fluctuations in their chemical composition due to environmental and agronomic factors, including crop type, soil conditions, and harvest season [154]. This pronounced compositional variability causes significant heterogeneity, ultimately compromising the predictability and quality parameters required for continuous industrial application. Also, extrusion machines typically require input with a moisture range of 10% to 30% [155]. VBPs contain a high moisture content, which requires high energy to dry large amounts of vegetable pomace or pulps, such as tomato pomace (which exhibits a moisture content of up to 90%) or carrot pomace (approximately 85%) [49,50].

Within the framework of a circular economy, the sustainability of biodegradable films derived from VBPs must be evaluated beyond raw material valorization, incorporating LCA, carbon footprint, and solvent recovery considerations. LCA provides a comprehensive approach to quantify environmental impacts across the entire value chain, including raw material collection, extraction, processing, film formation, and end-of-life scenarios [156]. Environmental performance is strongly influenced by processing steps, particularly extraction and solvent use. Green extraction technologies such as UAE and MAE contribute to lower energy consumption and reduced processing times compared to conventional methods, thereby decreasing the carbon footprint of the system. Among these, UAE is often associated with lower operational energy requirements due to its ability to operate at moderate temperatures, whereas MAE may involve higher energy input despite shorter processing durations [157]. Solvent selection and recovery also play a critical role in determining sustainability outcomes. The use of food-grade, low-toxicity solvents, such as ethanol–water mixtures, aligns with green chemistry principles [158]. However, without efficient recovery systems, solvent losses can increase both environmental impact and operational costs [159]. Integration of solvent recycling technologies, such as distillation or membrane-based recovery, can substantially improve process sustainability by reducing solvent consumption, emissions, and overall life cycle impacts [160,161]. End-of-life considerations, including biodegradability and compostability, further strengthen the circularity of these materials.

## 5. Regulatory Framework and Food Contact Compliance

Biodegradable films developed from VBPs must comply with the same food contact regulations as conventional food packaging if they are intended for direct food contact. In the European Union, food contact materials are regulated under Regulation (EC) No. 1935/2004, which requires that packaging materials must not transfer constituents to food in quantities that could endanger human health, alter food composition, or adversely affect its taste or odour. In addition, food contact materials must be manufactured according to Good Manufacturing Practice (GMP) under Commission Regulation (EC) No. 2023/2006. New substances used in food packaging may also require safety evaluation and authorization by the European Food Safety Authority before commercialization [162].

In the United States, food contact materials are regulated by the U.S. Food and Drug Administration. Components of biodegradable films must either be authorized under the relevant provisions of the Code of Federal Regulations, qualify as Generally Recognized as Safe (GRAS), or be approved through the Food Contact Notification (FCN) program before they can be legally marketed for food-contact applications [163].

## 6. Characterization of Film Properties

The characterization of films derived from VBPs is important for understanding their potential as sustainable resources for biodegradable packaging. These by-products are rich in diverse biopolymers such as polysaccharides (e.g., cellulose, pectin, starch), proteins, and bioactive compounds. Evaluation of film properties helps determine how effectively these components contribute to film performance.

Mechanical parameters, including tensile strength, elongation at break, and Young’s modulus, are commonly assessed to evaluate the structural integrity of films developed from vegetable residues [65,131]. These properties determine the ability of films to resist deformation and tearing during storage and transport when used as packaging material [164]. These properties are strongly influenced by the composition and interactions of the naturally occurring biopolymers within the by-products [165]. For example, incorporating potato peel waste into thermo-processed film matrices can provide strong structural integrity [166]. Polysaccharide-based carrot pomace films display excellent macro-structural flexibility, achieving good elongation at break percentages [135].

Measuring barrier properties is essential to determine how effectively the material protects its contents from the environment. As biopolymers are naturally sensitive to moisture and gases, characterizing these transfer pathways is a critical step in material design [167]. Barrier properties, including WVP, oxygen permeability (OP), light, and UV barrier properties, are mainly evaluated [65,131]. Determining water-vapor permeability is important because water can penetrate through films, leading to deterioration in the quality of food products [131]. Due to the hydrophilic nature of many plant-derived polymers, films often exhibit poor moisture resistance [168]. The abundance of free hydroxyl (-OH) and polar groups in natural materials that readily interact with water results in high water-vapor permeability and poor film integrity under humid conditions [169,170]. Therefore, characterization in this context helps identify the need for modifications such as introducing hydrophobic agents and other active constituents [168]. For example, Borah et al. [65] reported that low- or intermediate-moisture foods are more suitable for the application of potato peel-based films than high-moisture foods, stating that slightly higher potato peel content has a negative impact on the moisture absorption rate. Xie et al. [131] incorporated bacterial cellulose into biodegradable films developed with a potato-peel-based biopolymer matrix and reported that interactions between bacterial cellulose and biopolymers with hydrogen bonding led to a decrease in the number of hydrophilic free hydroxyl groups and thus a decrease in WVP of the films. Measuring OP is critical because oxygen is a primary driver of food degradation [171]. OP measures how easily oxygen molecules can pass through the film over a specific period [172]. Merino et al. [45] reported that films developed from potato peel exhibited relatively high oxygen permeability, making them unsuitable for long-term storage of oxidation-sensitive foods. However, the authors suggested that these materials could still be effectively utilized for packaging dry food products, where oxygen barrier properties are less critical. The visible-light transmission properties of films are important as they directly influence the clarity and visibility of the packaged product. In the wavelength range of 400–800 nm, higher light transmission corresponds to greater transparency, which is desirable for consumer acceptance and product display. This property is typically evaluated using a UV-Vis spectrophotometer [135,173]. Borah et al. [65] reported that increasing the concentration of potato peel powder enhanced the transparency of the films, likely due to its hydrophobic nature. UV barrier properties are critical for protecting food products from light-induced degradation. In the wavelength range of 200–400 nm, lower light transmission indicates better UV-blocking ability. Exposure to UV light accelerates lipid oxidation and overall food deterioration [174]. Therefore, films with strong UV barrier properties are particularly suitable for packaging light-sensitive and lipid-rich foods, as they can effectively reduce photooxidative damage [175].

Structural and physicochemical analyses, including Scanning Electron Microscopy (SEM), X-ray Diffraction (XRD), and Fourier Transform Infrared Spectroscopy (FTIR), provide insights into the morphology and molecular interactions within the film matrix [139,176,177]. SEM provides detailed insights into surface morphology and cross-sectional structure. Due to the heterogeneous composition of these films, comprising polysaccharides, proteins, lipids, and insoluble fibers, variations in component distribution can significantly influence film uniformity. Akmeemana et al. [137] observed through SEM analysis that increasing the concentration of essential oil in cassava bagasse-enriched films led to greater heterogeneity within the film matrix. This was attributed to the formation of microcracks and structural discontinuities, which became more pronounced at higher essential oil levels. Authors further stated that such defects can propagate rapidly under mechanical stress, particularly during stretching, thereby adversely affecting the mechanical properties of the films. SEM images revealed that agglomerations occurred on the surface of the potato peel-based films with the addition of a high concentration of bacterial cellulose [73]. Chhoden et al. [139] observed that the incorporation of pomace extract at higher concentrations caused rough and uneven surfaces and suggested that the addition of fibers had hindered the binding of starch and hydroxymethyl cellulose, causing the disruption of bonds.

FTIR determines the chemical composition, molecular structure, compatibility, and miscibility between the biopolymers [37]. For example, FTIR results revealed that subjecting carrot pomace powder to thermomechanical processing caused changes in the hydrogen bonding or the molecular environment of the hydroxyl groups, along with an increment in the availability of hydroxyl groups in the sample [82]. The authors attributed the changes to processing and plasticization with water. In contrast, FTIR findings reported by Xie et al. [131] revealed that the formation of hydrogen bonds between the bacterial cellulose and the biopolymers in the potato peel matrix led to the formation of a compact structure of the films. These findings highlight that variations in intermolecular interactions can have differing effects on film performance; disruption of hydrogen bonding generally enhances chain mobility and flexibility but may weaken mechanical strength and barrier properties, whereas the formation of strong intermolecular interactions promotes structural integrity, resulting in improved mechanical strength and reduced permeability. XRD analysis is performed on the biodegradable films to evaluate their crystallinity [54,140]. The crystallinity of the films depends on the effect of processing and additives. The degree of crystallinity plays a critical role in determining water-vapor permeability [44]. Merino et al. [45] observed a decrease in crystallinity after the incorporation of plasticizers. Similarly, XRD patterns indicated a reduction in film crystallinity upon essential oil incorporation into degradable packaging films from cassava bagasse since water-vapor transmission in biodegradable films predominantly occurs through the amorphous regions of the polymer matrix [68].

Thermal properties, evaluated using differential scanning calorimetry (DSC) and thermogravimetric analysis (TGA), help determine the stability and processing suitability of the films [176]. DSC provides insights into key transitions, such as glass transition temperature (Tg) and melting temperature, which are associated with polymer mobility, crystallinity, and film flexibility [178]. On the other hand, TGA reveals thermal degradation patterns and weight-loss stages, enabling the assessment of compositional stability and decomposition behavior under increasing temperatures [179]. These thermal parameters are particularly important for understanding the effects of plasticizers, bioactive compounds, and processing treatments on film structure [180]. Thermal behavior is closely linked to functional properties such as mechanical strength, barrier performance, and storage stability, making it a critical factor in designing films for food packaging applications [152].

Characterizing the functional properties is essential to assess their role in extending shelf life and enhancing food safety. In this context, antioxidant activity and antimicrobial properties are commonly assessed to determine the film’s ability to inhibit oxidative reactions and suppress the growth of spoilage and pathogenic microorganisms [65,131]. Biodegradability is a critical parameter that reflects the environmental sustainability of these materials. Biodegradability tests assess the material’s ability to safely decompose into natural elements (carbon dioxide, water, and biomass) when exposed to microorganisms, moisture, and varying temperatures. These tests typically evaluate three primary environmental conditions: controlled composting, soil burial, and aqueous/marine exposure [135,181].

Overall, the characterization of films derived from VBPs not only evaluates their physical and functional performance but also provides critical insights into how these low-cost, renewable resources can be effectively utilized in sustainable packaging applications.

## 7. Future Recommendations

More research is needed on the direct utilization of whole VBPs rather than relying primarily on extracted biopolymers. This approach could reduce processing steps, minimize incompatibility between biopolymers, and improve cost-effectiveness. As highlighted in this review, most studies focus on isolated components, while the use of whole biomass remains limited.

Based on current research, some of the most promising yet underutilized sources, largely due to the substantial quantities generated and often discarded during industrial processing, include carrot pomace, tomato pomace, potato residues and sugar beet pulp. Pomace and pulp are generated as major side streams during the processing of root vegetables in industries such as juice production (e.g., carrot and beetroot), sugar manufacturing (sugar beet), and starch extraction (potato), accounting for approximately 25–50% of the total processed biomass [182]. Tomato pomace is abundantly available as a by-product of large-scale tomato processing industries, where significant quantities of peels, seeds, and residual pulp are generated during the production of juice, paste, and sauces, typically accounting for a substantial fraction of the processed raw material [183]. Approximately one quarter of the total annual tomato production is utilized for industrial processing, establishing tomatoes as the world’s leading vegetable for processing and generating an estimated 6.2 million tonnes of tomato pomace each year [184]. These residues are rich in structural polysaccharides (e.g., cellulose and pectin) and bioactive compounds (e.g., phenolics and carotenoids), which contribute to their film-forming ability and make them suitable for biodegradable film development. Therefore, future research should prioritize the effective utilization of these abundantly available residues as primary film-forming matrices.

Secondly, future studies should prioritize the efficient utilization of bioactive compounds naturally present in vegetable residues. Current research largely depends on externally added bioactive agents such as essential oils and plant extracts, while the inherent antioxidant and antimicrobial compounds in VBPs remain underexploited. Strategies to preserve and enhance these native bioactives during processing should be further explored.

Another important area is the optimization and scalability of green extraction technologies. Although techniques such as UAE, MAE, and EAE have shown promising results, challenges related to process scalability, cost, and operational efficiency must be addressed. Based on available research, UAE integrated with a DES can be recommended as the most suitable technology for the valorization of VBPs in biodegradable film applications. It provides an optimal balance between extraction efficiency, cost-effectiveness, scalability, and preservation of structural polymers and bioactive compounds. DES acts as both the extraction solvent and the functional film plasticizer simultaneously.

Additionally, more comprehensive life-cycle assessment (LCA) studies are required to evaluate the environmental impact of these extraction methods and solvent systems. The stability and functionality of bioactive compounds during film formation and storage remain insufficiently studied. Factors such as heat, light, oxygen exposure, and drying conditions can significantly affect the activity of these compounds. Therefore, future research could focus on improving stability through advanced incorporation techniques such as encapsulation, nanoemulsions, and multilayer film design. In addition, the improvement of barrier properties, particularly moisture and oxygen resistance, remains a major challenge for vegetable-based films due to their hydrophilic nature.

Beyond food packaging, the functional properties of VBP-derived films present opportunities for a range of emerging applications. In the biomedical field, these films could potentially serve as biodegradable wound dressings, where their antioxidant and antimicrobial properties may enhance wound healing and reduce the risk of infection. However, their use in such applications will require comprehensive evaluation of biocompatibility, sterility, mechanical performance, and overall safety. Additional potential applications include biodegradable agricultural mulch films, flexible substrates for electronic devices, and sustainable packaging for cosmetic and apparel products. Their water-soluble and biodegradable characteristics also make them promising candidates for single-use packaging applications, such as cosmetic and personal care samples, bath products, and unit-dose laundry detergent pods, where the packaging can dissolve completely in water or biodegrade safely after use, thereby reducing plastic waste.

Finally, greater consistency in methodologies for film characterization and performance evaluation would be beneficial to facilitate more meaningful comparisons across studies. Consistent reporting of parameters such as barrier properties, mechanical strength, and functional activity will support the advancement of this field.

## 8. Conclusions

VBPs hold significant potential as low-cost, renewable resources for the development of sustainable and functional biodegradable films. Advancing this field will require integrated efforts in (i) optimizing extraction processes, (ii) improving film performance, (iii) enhancing the stability and effectiveness of bioactive compounds, (iv) utilizing naturally occurring bioactives within VBPs to develop inherently functional films, thereby reducing the need for external additives, (v) lowering production costs, and (vi) scaling up production technologies. With continued research and innovation, vegetable by-product-based films can play a crucial role in supporting circular bioeconomy strategies and transitioning toward more sustainable food packaging solutions.

## Data Availability

No new data were created or analyzed in this study. Data sharing is not applicable to this article.

## Abbreviations

The following abbreviations are used in this manuscript:

VBP	Vegetable by-products
EAE	Enzyme-assisted extraction
UAE	Ultrasound-assisted extraction
DES	Deep eutectic solvents
ILs	Ionic liquids
MAE	Microwave-assisted extraction
SFE	Supercritical fluid extraction
LCA	Life-Cycle Assessment
SEM	Scanning Electron Microscopy
FTIR	Fourier Transform Infrared Spectroscopy
TGA	Thermogravimetric analysis
DSC	Differential scanning calorimetry
Tg	Glass Transition Temperature
XRD	X-ray Diffraction
PVA	Polyvinyl alcohol
WVP	Water Vapor Permeability
TCC	Total carotenoid content
SCCO_2_	Supercritical CO_2_
SFE	Supercritical fluid extractions
RSM	Response Surface Methodology
MCC	Microcrystalline cellulose
